# Wisconsin Society of Veterinary Graduates

**Published:** 1897-03

**Authors:** W. G. Clark

**Affiliations:** Secretary


### SOCIETY PROCEEDINGS.

---

### WISCONSIN SOCIETY OF VETERINARY GRADUATES.

THE annual meeting was held in the City Hall at Madison, February 5, 1897. Meeting called to order by the President, Dr. Leech, at 2 P.M. Roll-call: Drs. Arpke, B. L. Clark, W. G. Clark, Hartwig, Kuoni, Leech, Laws, Ormond, E. D. Roberts, Roub, and L. A. Wright present. Dr. A. H. Wright, of Lake Mills, was also present.

Minutes of last meeting read and approved. The Secretary's report was read and accepted. The Treasurer's report was read and accepted. Dr. Leech then gave his annual address, which was very interesting and instructive, and contained a great deal of good advice to the practitioner. On motion, a vote of thanks was tendered Dr. Leech for his able address.

The following addition to the code of ethics was discussed, and, on motion, adopted: "It shall be deemed a violation of the code of ethics for any member of this Society to contract with or through the officers of any live-stock insurance company for the professional treatment of the members' stock so insured, but this rule shall not prevent any member from becoming the examiner of risks and to act in the capacity of an expert for the same."

The application of Dr. S. S. Snyder, of Cedarburg, was then presented for membership. It was referred to the Board of Censors, while the Society took a short recess. The Censors reporting favorably, it was moved and seconded that Dr. Snyder be elected to membership; carried.

Dr. Leech, as Chairman of the Committee on Legislation, reported that a bill to regulate the practice of veterinary medicine and surgery had been drafted and was ready for presentation to the Legislature, and gave the important parts of the same. The bill was discussed by Roberts, Hartwig, Ormond, Clark, and Wright. On motion, the report of the committee was accepted. It was moved and seconded that the Secretary send each member a copy of the bill when printed; carried.

The Secretary read a letter from Dr. Salmon requesting a contribution to the Pasteur Memorial Fund. On motion, the Secretary was instructed to send five dollars.

The Secretary reported correspondence in regard to the army veterinary bill. On motion, further correspondence in regard to army veterinary legislation was left to the Secretary.

Dr. Oberst being absent on account of sickness, the Secretary read his paper on "Tuberculosis."

On motion, the Society adjourned to meet at 7 P.M.

### Evening Session.

On motion, the regular order of business was dispensed with, and the Society proceeded to the election of officers. The following gentlemen were elected for the ensuing year: President, Dr. L. A. Wright, Columbus; Vice-President, Dr. J. F. Roub, Monroe; Secretary, Dr. W. G. Clark, Beaver Dam; Treasurer, Dr. J. P. Laws, Madison; Censors, Drs. A. H. Hartwig, Watertown; B. L. Clark, Monticello; and E. H. Newton, Waupun.

The reading of papers was then taken up. Dr. B. L. Clark read an interesting and carefully prepared paper on "Laminitis." Discussed by Drs. Ormond, Hartwig, Wright, Roberts, and Roub.

Dr. Arpke read a paper on "Reflex Irritation," citing a number of cases. It was very interesting, and brought out plainly the necessity of a careful examination in order to arrive at a correct conclusion as to the causes of disease in many cases. Discussed by Drs. Roberts, Wright, and Hartwig.

Dr. J. F. Roub reported a case of "Lipoma causing Death." The case was diagnosed at first as enteritis. Post-mortem revealed a lipomatous tumor on the posterior aorta. Discussed by Drs. Ormond, Wright, and Laws.

Dr. J. P. Laws reported several interesting cases that had occurred in his practice. Discussed by Drs. Hartwig and Roberts.

On motion, it was decided to hold the semi-annual meeting in Milwaukee, subject to the call of the Secretary.

On motion, a vote of thanks was tendered the essayists.

Adjournment. W. G. Clark, M.D.C.,
Secretary.

